# Novel Applications of Lead Acetate and Flow Cytometry Methods for Detection of Sulfur-Containing Molecules

**DOI:** 10.3390/mps2010013

**Published:** 2019-02-01

**Authors:** Evgeniya Anishchenko, Carmela Vigorito, Luigi Mele, Patrizia Lombari, Alessandra F. Perna, Diego Ingrosso

**Affiliations:** 1 Department of Translational Medical Sciences, University of Campania “Luigi Vanvitelli,” 80131 Naples, Italy; anishchenkoea@gmail.com (E.A.); ca.vigorito86@libero.it (C.V.); patrizia.lombari@gmail.com (P.L.); 2 Department of Precision Medicine, University of Campania “Luigi Vanvitelli,” 80138 Naples, Italy; diego.ingrosso@unicampania.it; 3 Department of Experimental Medicine, University of Campania “Luigi Vanvitelli,” 80138 Naples, Italy; luigi.mele@unicampania.it

**Keywords:** H_2_S, SSP4, fluorescent probe, sulfane sulfur species, lead acetate test strips, flow cytometer, fluorescence imaging, agar trap, cell culture

## Abstract

Hydrogen sulfide (H_2_S) is the most recently established gaseous vasodilator, enzymatically produced from cysteine metabolism, involved in a number of pathophysiological processes. However, its accurate detection in vivo is critical due to its volatility and tendency to form sulfane sulfur derivatives, thus limiting the data interpretation of its biological roles. We developed new applications of the simple and rapid method to measure H_2_S release in cell culture systems, based on the lead acetate strip test. This test, previously prevalently used in microbiology, was compared with the agar trap method, applied, in parallel, on both cell cultures and cell-free samples. Sulfane sulfur represents the major species derived from intracellular H_2_S. Various fluorescent probes are available for quantitation of H_2_S derivatives intracellularly. We present here an alternative to the classic imaging method for sulfane sulfur evaluation, running on a flow cytometer, based on SSP4 probe labeling. Flow cytometry turned out to be more direct, fully quantitative and less time-consuming compared to microscopy and more precise with respect to the fluorescence multi-plate reader assay. The new application methods for H_2_S determination appear to be fully suitable for the analysis of H_2_S release and sulfane sulfur content in biological samples.

## 1. Introduction

Hydrogen sulfide (H_2_S), previously only considered a hazardous gas, was, in the 1990s, detected in mammalian tissues and found indispensable in physiological concentrations. H_2_S is biosynthesized through enzymatic reactions in human tissues, although 50% of this gas is generated by the microbiota, in the inner and outer mucus layers of the intestine [1]. In the last two decades, the metabolism and physiological roles of H_2_S and its derivatives, such as the amount released from Fe-S proteins and sulfane sulfur species, have been also related to various pathological situations [2,3]. Controversies have been raised on the physiological H_2_S active concentrations, which have been detected in the 10^−5^−10^−4^ molar range [4,5]. Detailed study of H_2_S role in metabolic processes showed its beneficial effects on neuromodulation [6], cardiovascular (CV) [7,8,9] and immune systems, while its impairment was observed in several pathologies such as renal dysfunction [3], atherosclerosis [10,11], diabetes [12] and hypertension [13]. 

Two major fractions of metabolically active H_2_S have been characterized and are generally worth evaluating: free H_2_S and sulfane sulfur [1,14,15,16]. Cell model systems are largely employed for studies on vasodilator gases, including H_2_S. In these subsets, measurements of free H_2_S can be attained using the agar trap method, which is based on the methylene blue reaction [17]. Other colorimetric methods, based on reactions with silver ions [18], bismuth (III) chloride [19] and potentiometric methods [20,21,22], were developed to detect H_2_S production. These last methods generally are time- and work-consuming procedures and/or their accuracy has been criticized. On the other hand, we were intrigued from acknowledging that the lead acetate strips test, used to detect H_2_S produced by microorganisms [23] and to evaluate the quality of water and food [24,25], although declared by the producer “*the more sensitive than any other method for detecting hydrogen sulfide production*” [23], has never been used, for quantitating H_2_S release in cell cultures, on a routine basis. We have indeed here developed a new protocol for the application of the lead acetate strip test to cell cultures. 

Hydrogenous polysulfides are actual signaling molecules with even higher nucleophilicity and reducibility than H_2_S [26]. The fluorescence probes for hydrogenous polysulfides achieved widespread applicability in recent studies [27,28,29,30,31]. Some of these probes are even organelle-specific and thus able to selectively label and allow tracing sulfur compounds in mitochondria [26,32]. Moreover, it is possible to monitor, in real-time, variations in polysulfide cell content and/or in vivo [33]. Among a vast variety of fluorescence probes, potentially able to interact with sulfur species, endogenous hydropolysulfides, H_2_S, etc., we chose the widely used and commercially available Sulfane Sulfur Probe 4 (SSP4).

Sulfane sulfur is a very important species, including persulfides (R-S-SH), polysulfides (R-S_n_-SH or R-S-S_n_-S-R), inorganic hydrogen polysulfides (H_2_S_n_, n ≥ 2) and protein-bound elemental sulfur (S^8^). Sulfane sulfur related species are generated following oxidation of cellular H_2_S and they are thought to serve as a source of endogenously released H_2_S [14,34]. To measure sulfane sulfur, several specific molecular probes have been developed [35,36]. In this work, we also successfully employed cytofluorometry, coupled with molecular labeling using the last generation of SSP4, to measure sulfane sulfur production in cell cultures.

## 2. Experimental Design

**Molecular mechanisms involved in H_2_S production and regulation**

Cystathionine-β-synthase (CBS) and Cystathionine-γ-lyase (CSE), are transsulfuration pathway enzymes, which independently catalyze H_2_S production intracellularly [37,38]. *S*-adenosyl-methionine (SAM) is a powerful allosteric activator of CBS; vitamin B6 is the precursor of the pyridoxal phosphate coenzyme for both CBS and CSE. Cysteine (Cys) is the major CBS and CSE substrate for H_2_S biosynthesis [38]. Aminooxyacetic acid (AOAA) and DL-propargylglycine (PAG) are CBS and CSE inhibitors, respectively, which were used to assess H_2_S enzymatic production. Diallyl trisulfide (DATS) is a H_2_S donor which rapidly releases H_2_S in the presence of Cys or glutathione (GSH) [39]. Polysulfide compounds, such as DATS, require an excess of Cys to release H_2_S in a cell-free environment [40], while DATS is able to release H_2_S into the cell without an excess of Cys, due to endogenous presence of this amino acid [41]. Hence, cells treated with DATS and Cys should also increase H_2_S release independently from enzymatic production. In our experiments, we also added a sample treated with enzyme inhibitors to dissect the contribution of enzymatic biosynthesis from that of chemical release.

### 2.1. Detection of Released H_2_S Using Agar Trap or Lead Acetate Strip Test (LAST)

The aim of this part of the experimental design was to set the conditions for the use of the lead acetate strip test (LAST) to quantitate H_2_S release from cell cultures. In this phase, indeed, LAST assays were performed in parallel with another well-established test usually employed to evaluate the release of H_2_S in vitro. The LAST assay is based on the following reaction:

Pb(CH_3_COO)_2_ + H_2_S → PbS + 2CH_3_COOH

where the formation of PbS as a black precipitate monitors the reaction completion. 

Conversely, we use the agar trap colorimetric assay, as a reference method for H_2_S detection. This method is based on the entrapping of S^2−^ by reaction with zinc acetate in a gel matrix. Then, the assay detects methylene blue, formed as the result of the reaction of S^2−^ released from the matrix with N,N-Dimethyl-p-phenylenediamine sulfate in the presence of FeCl_3_ in an acidic microenvironment.

First, we prepared the reference standard for construction of the calibration curve. To this purpose, serial dilutions of NaHS solution in the 25–200 μM range were prepared to obtain standard curves in a concentration range suitable for both methods and to compare the two methods in the concentration range of interests (Figure 1a).

Then, we performed parallel experiments using agar trap and LAST to evaluate H_2_S, released from cell cultures in time courses at 6 h and 12 h. The H_2_S evaluation was performed on five experimental groups and contained: (1) non-treated control CTRL (−); (2) stimulated control, CTRL (+): Cys, B6, and SAM; (3) inhibitors (PAG and AOAA); (4) stimulators (Cys, B6, and SAM) and inhibitors (PAG, AOAA); and (5) DATS and stimulators (Figure 1b). 

Lastly, we performed a 1 h experiment using the agar trap method with cell-free 100 and 200 μM NaHS aqueous solutions in different humidity conditions (36 and 68%) to demonstrate the influence of the microenvironment onto the preciseness of agar trap method (Figure 1c). 

### 2.2. Measuring of Sulfane Sulfur Species Using SSP4 and Flow Cytometry in Comparison to Fluorescence Microscopy

The aim of this part of the experimental design was to optimize the use of SSP4 as a fluorescence probe to quantitate sulfane sulfur intracellularly. SSP4 is a fluorescent probe of the last generation, capable of reacting with sulfane sulfur species, according to the reaction scheme in Figure 2a, thus allowing quantitative detection with high accuracy, reproducibility and sensitivity. Fluorescence microscopy and spectrometry (including multi-plate readers) are the recommended techniques to detect SSP4 labeling, as suggested by the supplier’s protocol. Our aim was to optimize this technique, by increasing precision and dependability of this approach. We chose flow cytometry to evaluate sulfane sulfur content in live cells using SSP4, in parallel fluorescence microscopy for comparison (Figure 2b). In addition to the experimental groups described in Section 2.1, we accomplished these experiments under various conditions. We included in the protocol cell samples treatments with glutathione (GSH) and stimulators (Cys, B6, and SAM), which are expected to increase sulfane sulfur formation. In fact, it was demonstrated that activation of CBS/CSE, in presence of GSH and Cys, generates CysSSH and CysSSSH, subsequently forming sulfane sulfur species due to sulfur transfer from CysSSH to GSH in a CBS/CSE dependent manner [42].

### 2.3. Materials

- Agar (AppliChem, Darmstadt, Germany; Cat. no.: A0949, 1000)
- Aminooxyacetic acid hemihydrochloride, AOAA (Aldrich, St.Louis, MO, USA; Cat. no.: C13408)
- Cetyltrimethylammonium bromide, CTAB (Sigma, St.Louis, MO, USA; Cat. no.: H9151)
- Diallyl trisulfide, DATS (Cayman Chemical, Ann Arbor, MI, USA; Cat. no.: 2050-87-5)
- Dimethyl sulfoxide, DMSO (Sigma-Aldrich, St.Louis, MO, USA; Cat. no.: W387520)
- DL-cysteine, Cys (Aldrich, St.Louis, MO, USA; Cat. no.: 861677)
- DL-propargylglycine, PAG (Sigma, St.Louis, MO, USA; Cat. no.: P7888)
- Dulbecco’s Modified Eagle’s Medium, DMEM (Sigma, St.Louis, MO, USA; Cat. no.: D4947)
- Dulbecco’s Phosphate-Buffered Saline, PBS (Microtech Srl, Pozzuoli, Italy; Cat. no.: L0615-500)
- Fetal Bovine Serum (South America), FBS (Microtech Srl, Pozzuoli, Italy; Cat. no.: S1810-500)
- Human Endothelial Somatic Cell Hybrid, EA.hy926 (American Type Culture Collection, Manassas, VA, USA; Cat. no.: CRL-2922^TM^)
- Hydrochloric Acid, HCl (Sigma, St.Louis, MO, USA; Cat. no.: H1758)
- Iron (III) chloride, FeCl_3_ (Sigma-Aldrich, St.Louis, MO, USA; Cat. no.: 157740)
- Lead acetate strips test (Sigma-Aldrich, St.Louis, MO, USA; Cat. no.: 06728)
- L-glutamine (Microtech Srl, Pozzuoli, Italy; Cat. no.: X0550-100)
- L-glutathione reduced, GSH (Sigma, St.Louis, MO, USA; Cat. no.: G6013)
- *N*,*N*-dimethyl-p-phenylenediamine sulfate salt, NDPS (Aldrich, St.Louis, MO, USA; Cat. no.: 186384)
- Penicillin–streptomycin (Microtech Srl, Pozzuoli, Italy; Cat. no.: L0022-100)
- Pyridoxine hydrochloride, B6 (Sigma, St.Louis, MO, USA; Cat. no.: P6280)
- *S*-adenosyl-methionine, SAM (New England Biolabs Inc, Ipswich, MA, USA; Cat. no.: B9003S)
- Sodium hydrosulfide hydrate, NaHS (Sigma-Aldrich, St.Louis, MO, USA; Cat. no.: 161527)
- Sodium hydroxide pellets, NaOH (Carlo Erba, Rodano, Italy, Cat. no.: 480507)
- Sulfane Sulfur Probe 4, SSP4 (Dojindo Laboratories, Kumamoto, Japan; Cat. no.: SB10)
- Trypsin-EDTA (Microtech Srl, Pozzuoli, Italy; Cat. no.: L0940-100)
- Zinc acetate dihydrate, ZnAc (Sigma, St.Louis, MO, USA; Cat. no.: 383058-500G)

### 2.4. Equipment

- Multimode Microplate Reader “Tecan Infinite” (Tecan Group Ltd, Männedorf, Switzerland; Cat. no.: M200)
- Confocal Laser Scanning Microscope (Zeiss, Milan, Italy; Cat. no.: LSM 710)
- Flow cytometer Accuri™ C6 Plus (BD Biosciences, San Jose, CA, USA)
- UV/VIS Spectrophotometer Lambda 25 (Perkin Elmer, Waltham, MA, USA; Cat. no.: L6020060)
- Multifunctional InkJet printer (Brother Industries, Ltd, Nagoya, Japan, Cat. no.: DCP-197C)
- Water Jacketed Incubator Thermo Forma 3851 (Thermo Fisher Scientific, Waltham, MA, USA)
- Incubator Heraeus B6 (Kendro, Langenselbold, Germany, Cat. no.: 63505)
- Laboratory sterile hood (STERIL spa, Milan, Italy, Cat. no.: VBH 48 MP/99)
- Centrifuge (Eppendorf, Hamburg, Germany, Cat. no.: 5810 R)

## 3. Procedure

**Cell culture and treatments**

Grow human cell line EA.hy926 in DMEM medium with 10% FBS, 2 mM L-glutamine and 0.1% penicillin–streptomycin. For H_2_S release detection experiments, plate cells in cell culture flasks 14 h before any experiment. Cell treatments include:

- 5 mM Cys, 5 mM B6, 10 μM SAM;
- 2 mM GSH;
- 150 μM DATS; and
- 2 mM PAG, 2 mM AOAA.

Cells growth conditions: 37 °C in a humidified atmosphere with 5% CO_2_.

Perform LAST and agar trap experiments, in triplicates in two biological repeats. Each sample consist of 500,000 cells in 5 mL of DMEM left overnight before treatment in 25 mL cell culture flasks. Standard treatment duration is 6 h and 12 h, respectively, under the above conditions. 

### 3.1. LAST in Comparison to Agar Trap Method. Time for Completion: Four Days

#### 3.1.1. Agar Trap Method

**Figure 3:**
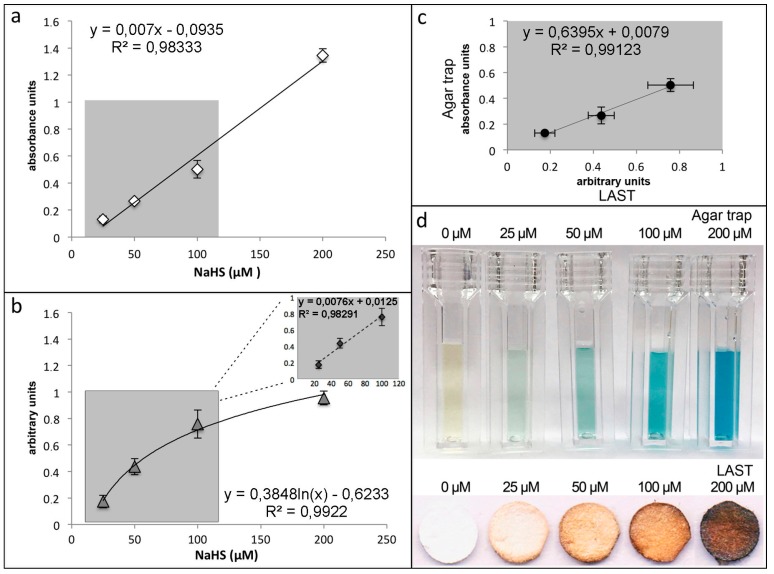
Comparative analysis of efficacy of agar trap and LAST: (**a**) calibration curve of NaHS using agar trap; (**b**) calibration curve of NaHS using LAST where inset (dashed line) shows the calibration curve in the 25-100 μM NaHS concentration range; (**c**) comparison of the NaHS calibration curves obtained using LAST (*x*-axis) and agar trap (*y*-axis) methods in the 25–100 μM NaHS concentrations range; and (**d**) both photographs show reaction products obtained of H_2_S, detected with agar trap developed by methylene blue staining (top), or lead acetate treated filters (bottom). LAST, lead acetate strip test.

**Figure 4:**
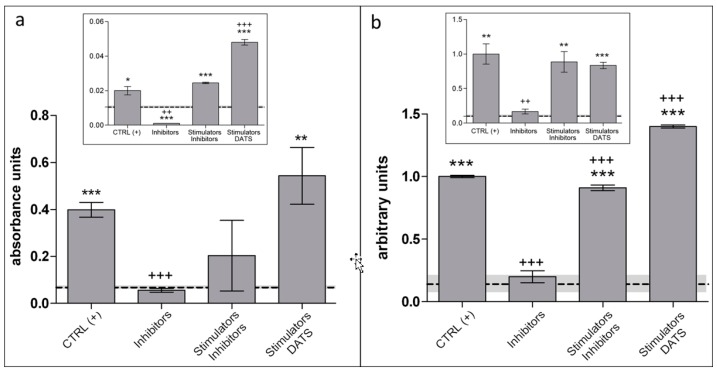
H_2_S release in 6 h and 12 h duration assay by human endothelial cells: (**a**) levels of H_2_S detected by agar trap method in 6 h and in 12 h (inset); and (**b**) levels of H_2_S detected by the LAST method in 6 h and 12 h (inset). CTRL (+)/Stimulators; 5 mM Cys, 5 mM vitamin B6, 10 μM SAM. Inhibitors; 2 mM DL-PAG and AOAA. (a,b) Samples are compared to the untreated controls CTRL (−) (indicated by the dashed baseline, SD is depicted by a gray band; data are illustrated as the mean ± SD; p value versus untreated controls: * *p* < 0.05, ** *p* < 0.01, *** *p* < 0.001, while *p* value versus CTRL (+): ^++^ *p* < 0.01, ^+++^ *p* < 0.001 (according to Student’s two-tailed *t*-test).

**Figure 5:**
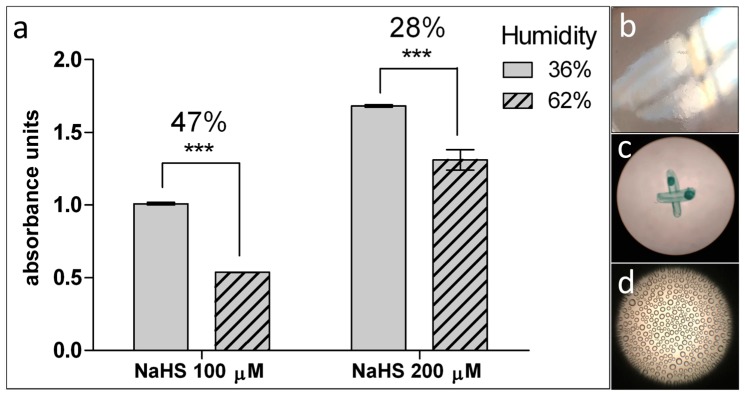
Effect of microenvironment humidity on the H_2_S recovery in the agar trap assay. The detection of H_2_S release is performed with agar trap method. (**a**) H_2_S released from 2 mL 100 and 200 μM NaHS aqueous solution, in 1 h at 37 °C by incubating in 36% or 62% humidified atmosphere. Percentage difference between samples incubated under various microenvironment conditions are indicated above each histogram. Examples of: (**b**) Photograph of polymerized dry agar gel before experiment; (**c**) microphotograph of agar gel in the sample incubated at 36% of humidity; and (**d**) microphotograph of agar gel with microdrops in a sample incubated at 62% of humidity. *p* value versus sample incubated at 36% of humidity: *** *p* < 0.001 (according to the Student’s two-tailed *t*-test).

Prepare flasks for agar trap method as described before [3,17]. Flasks are laid horizontally upside-down; 5 mL of 1% agar with 7.23 mM ZnAc and 162 mM NaOH are allowed to polymerize on the bottom surface inside each flask. Once the agar is solidified, turn the flasks and plate cells on the bottom. Treat cells as appropriate. At the end of treatment, withdraw the medium and lay 2 mL of 500 mM NaOH, 100 μM EDTA solution onto the agar gel and right after 500 µL of detection solution (17.1 mM NDPS and 37 mM FeCl_3_ in 6 M HCl) [43]. Measure color development in 15 min with spectrophotometer at λ668 nm against an agar gel blank kept with cell free DMEM (Figure 3a,d, Figure 4a and Figure 5).

#### 3.1.2. LAST

##### Standard Curve. Time for Completion: 02:45 Hours

1. Cut lead acetate strips using a paper perforator;
2. Place test strips into the internal side of each flask lid, using a double-sided adhesive tape.
3. Sterilize flask lids using UV light.
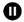
**PAUSE STEP** Sterile lids can be stored at 4 °C until use for weeks.
4. For standard curve, add 3 mL aqueous solution containing 25, 50, 100, and 200 μM NaHS to flasks and tightly close with the strip containing lids, following incubation for 1 h at 37 °C.
5. At the end of the experiment, place test strips on Whatman paper sheets, and scan using a multifunctional printer.
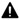
**CRITICAL STEP** Scanning should be performed within 24 h after experiment, since, within 3–5 days after experiment, the color can fade away.
6. Image analysis is accomplished using ImageJ software (National Institutes of Health, Bethesda, MD, USA): Analyze > Measure and Analyze > Histogram. Set fixed reading slit for each series of assay measurements (see Figure 3b,d).

##### LAST in Cell Culture. Time for Completion: Four Days

1. Treat cell culture in flasks as described above and tight lids containing test strips. Incubate for 6 h and/or 12 h in 5% CO_2_ atmosphere.
2. At the end of experiment, repeat Steps 5 and 6 as in the previous paragraph.
3. Data are analyzed with ImageJ software, using GraphPad Prism 5 for statistics (GraphPad Software, La Jolla, CA, USA) and applying Student’s two-tailed *t*-test (Figure 4b).

### 3.2. Sulfane Sulfur Quantitation with SSP4 Using Flow Cytometry in Comparison to Fluorescence Microscopy. Time for Completion: 00:45 Hours 

Plate 60 mm petri dishes with 500,000 cells, in 3 mL of DMEM and leave overnight in incubator, before the experiment. Prepare enough dishes to perform each treatment for both fluorescence microscopy and flow cytometry in triplicate in two biological repeats, during 3 h. 

#### 3.2.1. Sulfane Sulfur Quantitation with SSP4 Using Fluorescence Microscopy

**Figure 6:**
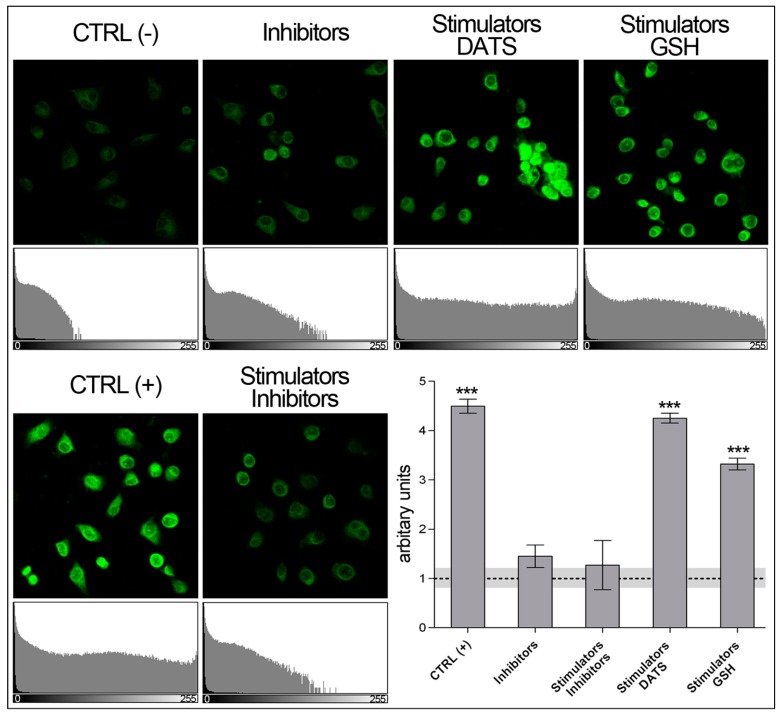
Detection of sulfane sulfur species using fluorescence microscope. Microphotographs of endothelial cells and corresponding histograms (below each microphotograph) show different sulfane sulfur-related fluorescence intensity, emitted by SSP4, in 3 h, with various treatments. Images analysis performed using ImageJ software and represented using arbitrary units (lower left diagram in each panel). Untreated control, CTRL (-), in diagram is indicated as dashed baseline; gray band is the relevant SD. Data depicted as the mean ± SD; p value versus the untreated control: ** *p* < 0.01, *** *p* < 0.001 (according to Student’s two-tailed *t*-test). CTRL (+)/Stimulators; 5 mM Cys, 5 mM vitamin B6, 10 μM SAM. Inhibitors; 2 mM DL-PAG and AOAA.

Follow the general directions reported in the technical manual provided by the supplier of the SSP4 fluorescent probe [44]. Briefly, after 3 h treatment, wash each cell sample twice with PBS and incubate for 15 min in 2 mL of FBS-free DMEM, with 10 μM SSP4 and 500 μM CTAB. Subsequently, wash once again and leave in PBS for imaging. SSP4-labeled cell imaging is captured using a confocal laser scanning microscope at λ_ex_482 nm and λ_em_515 nm. For data analysis, employ the ImageJ software (Figure 6).

#### 3.2.2. Sulfane Sulfur Quantitation Using SSP4 Labelig and Detection by Flow Cytometry

**Figure 7:**
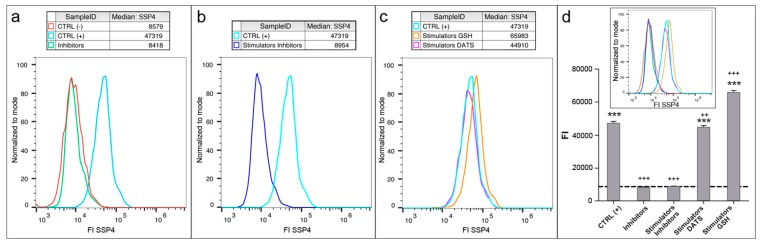
Detection of sulfane sulfur species using flow cytometry: (**a**–**c**) Spectrum of SSP4 fluorescence intensity, emitted in presence of sulfane sulfurs, in endothelial cells treated for 3 h. (**d**) FI median values are drawn in the histogram plot, where untreated control, CTRL (−), is indicated as a dashed baseline. (**d**) Inset is a merge of graphs in (**a**–**c**) to allow easier comparison of the relevant data. Data are depicted as mean ± SD; *p* value versus untreated control: *** *p* < 0.001, while *p* value versus CTRL (+): ^++^ *p* < 0.01, ^+++^ *p* < 0.001 (according to Student’s two-tailed *t*-test). CTRL (+)/Stimulators; 5 mM Cys, 5 mM vitamin B6, 10 μM SAM. Inhibitors; 2 mM DL-PAG and AOAA. FI; fluorescence intensity. Color code is the same in (**a**–**c**) and the inset of (**d**).

**Figure 1:**
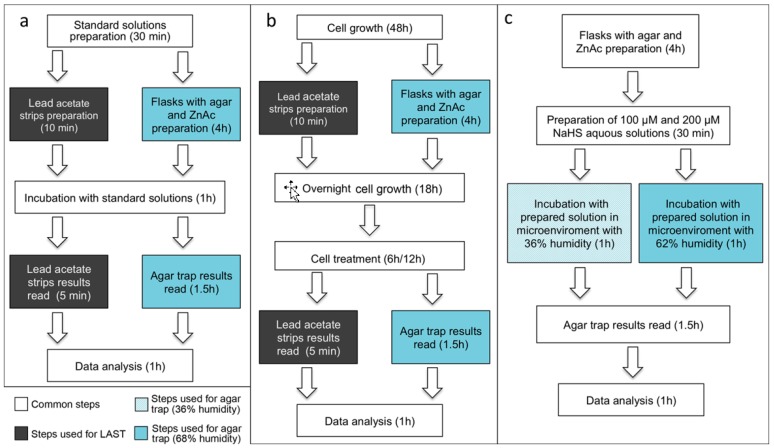
The scheme of experimental design for agar trap and LAST assays: (**a**) experimental design for standard curves; (**b**) scheme for experiments in cell culture; and (**c**) experimental design for detect levels of agar trap method precision in different microenvironmental conditions. ZnAc; zinc acetate.

1. Treatment duration is 3 h, then wash the cells twice with PBS and leave for 15 min in 2 mL of FBS-free DMEM with 1.5 μM SSP4 and 150 μM CTAB.
2. Wash twice again with PBS and leave for 3 min in the presence of 0.5 mL Trypsin-EDTA at 37 °C.
3. Transfer detached cell samples in 1.5 mL micro test tubes and wash once again with PBS.
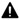
**CRITICAL STEP** The subsequent analysis should be performed within 5–15 min.
4. Detect SSP4-emitted fluorescence using a flow cytometer (Accuri™ C6 Plus).
5. Analyze data using Accuri™ C6 Plus and GraphPad Prism 5 software (Figure 7).

## 4. Expected Results

The lead acetate strip test was successfully adapted to quantify H_2_S release in cell culture and, compared with agar trap, was equally sensitive, during the first hour of incubation. Standard curves, for the agar trap and LAST methods, showed linear or exponential dependence of the amount of H_2_S released as a function of NaHS concentration (Figure 3a,b, respectively). The sensitivity of the two methods appeared comparable. In these experiments, NaHS, used as H_2_S donor, was dissolved in an aqueous solution and, hence, released as H_2_S is measured. 

The estimated detection linear range for gaseous H_2_S, released in 1 h at 37 °C from an aqueous solution of NaHS, using LAST method falls within 3–3.5 μg and 56 μg of NaHS. In terms of concentration, this corresponds, in good approximation, to 10÷200 μM NaHS initially dissolved in aqueous solutions. For LAST, we used 4 mm Ø lead acetate treated filters. Thus, the minimal amount of sulfur containing solute to be detected was 0.28 μg to be absorbed onto 1 mm^2^ filter surface. Saturation was reached at 4.5 μg/mm^2^. Agar trap method displays similar performances compared to LAST with respect to the lower threshold sensitivity level. Conversely, saturation was reached at lower NaHS amount (about 45 μg), corresponding to 160 μM NaHS in solution. We figured out that sensitivity of LAST and agar trap methods could be further increased by 25%, by reducing surface area of the lead acetate filter and by reducing gel surface, thus optimizing detection solution volume, respectively.

On the other hand, according to Sitdikova and coworkers [45], a strict relationship exists between the initial NaHS concentration and that of H_2_S released from the NaHS donor, by plotting the NaHS donor vs. the H_2_S currently measured with an anion selective electrode. According to this extrapolation, it was possible to indirectly evaluate H_2_S by multiplying NaHS concentration for a 0.4 factor. Applying this algorithm, the relationship of H_2_S concentrations derived from NaHS donor was linear for both agar trap and LAST methods, up to about 40 μM H_2_S, corresponding to 100 μM NaHS. The established range of linear dependence is suitable for experiments with cell cultures. In fact, in previous work, our research group observed maximal H_2_S release from stimulated hepatocarcinoma cells (HepG2) in the 30–50 μM range [3]. Standard curves resulting from NaHS were reproducible and linear, R^2^ = 0.99 (Figure 3c), which allowed us to proceed with LAST analysis in cell culture.

The following analysis of H_2_S release from cell culture showed consistent results for agar trap and LAST methods within 6 h of the various cell treatments (see Figure 4). In both experiments, CTRL (+) and DATS with stimulators in cell culture significantly increased the amount of released H_2_S in comparison to intact samples (dashed line) or in presence of CBS/CSE inhibitors. However, the LAST method resulted in a more precise and less dispersed data, after statistical analysis. 

**Figure 2:**
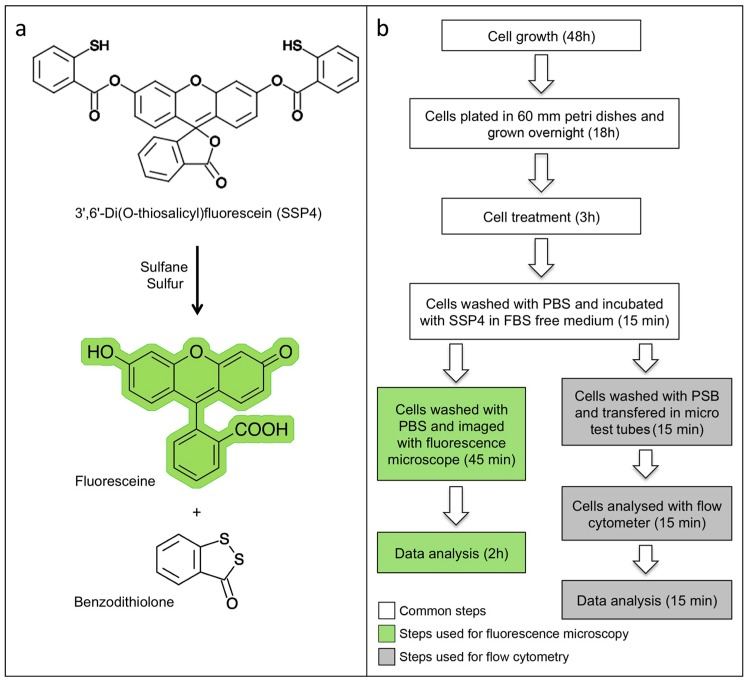
The principle of reaction and design of experiments with SSP4: (**a**) Sulfane Sulfur Probe 4 in presence of sulfane sulfur species cleaves fluorescein (shown in green) that could be detected at λ_ex_482 nm and λ_em_515 nm; and (**b**) experimental design for detection sulfane sulfurs in cells culture with fluorescence microscope and/or flow cytometer using SSP4. PBS, Phosphate-Buffered Saline; FBS, Fetal Bovine Serum.

Importantly, agar trap method seemed to diminish its sensitivity in experiments lasting longer than 6 h. We noticed 10-fold lower absorbance unit range in the 12 h experiment (inset in Figure 4a) in comparison to the experiment with 6 h incubation (Figure 4a). LAST and agar trap showed quite different results after longer cell exposures (12 h, insets in Figure 4). Noteworthy, the ratio of H_2_S levels, among samples detected by agar trap in 12 h, remained similar to that detected after 6 h (Figure 4a and inset in Figure 4a, respectively). DATS is a H_2_S donor that could induce saturation, while stimulators maintained CBS/CSE activity for much longer. Thus, levels of H_2_S released within 12 h in CTRL (+) and in DATS with stimulators become very similar, according to LAST (inset in Figure 4b), as opposed to results obtained with the agar trap method (inset in Figure 2a). We hypothesize that LAST, although capable of providing a result in a shorter incubation time, is also more suitable than the agar trap method to quantitate H_2_S release in long-lasting experiments (over 6 h), in that it does not appear to reach saturation within this time range. As shown in Figure 5a, the percentage of humidity significantly (≥ 28%) altered the agar trap sensitivity. It was noticed that agar layer in the flask with cell culture, in the CO_2_ incubator, became covered with condensation drops (Figure 5d). This did not occur in a lower humidity microenvironment (Figure 5c). Thus, the LAST method appears to be superior with respect to the agar trap, to measure H_2_S release from cell cultures, in that it insures: (a) higher stability over time; (b) less tendency to saturation of the detection system; and (c) does not appear to be crucially influenced by a high degree of humidity in the chamber. 

Both agar trap and LAST allow the detection of the total quantity of H_2_S released over time, during cell incubation in contrast to ultra-sensitive methods such as pre-column derivatization with monobromobimane and HPLC analysis (MBB/HPLC), able to detect H_2_S at nanomolar levels. For example, MBB/HPLC is a destructive method and does not allow the recovery of H_2_S extracted and entrapped in a filter throughout the duration of the experiment. We may say that the two methods are complementary, in the sense that LAST may provide a “rule of thumb” best approximation of H_2_S total release during incubation. Conversely, other more sensitive methods are suitable to detect end-point fine-tuned H_2_S release, provided that incubation time and experimental conditions are suitable to prevent massive evaporation of H_2_S, or other ways to entrap this gas are employed. 

Moreover, LAST does not need any special laboratory preparation or equipment. It is rapid, and cell samples are not consumed in the assay and can be recovered and used for further determination. The optimal range of released H_2_S that can be detected by LAST assay corresponds to H_2_S physiological concentrations found in mammals [4,5]. 

The common methods to quantify sulfane sulfur derivatives, employing fluorescent probes, are accomplished by fluorescence microscope imaging [35,42,46] or fluorescence spectrometry [14,36,42,47,48,49,50]. Fluorescence microscopy may achieve fully quantitative performances, by measuring fluorescence intensity (FI) using ImageJ or similar software. The spectrofluorometer (or multi-plate reader) also allows evaluating FI quantitatively, but this is performed on an approximate cell count based on the assumption of cell confluence in a certain plate surface. Sulfane sulfur detection, performed according to user’s manual supplied with SSP4, requires spectrofluorometer or multi-plate reader. Following the original protocol, we noticed the high divergence in quantity of cells which were lost during procedures. The alterations of cells adhesion and consequently their loss after washing procedures can be due to CTAB, a cationic surfactant hampering cell adhesion [51]. CTAB however is needed to transport SSP4 into the cells. We improved the original protocol, by reducing both SSP4 concentrations used, along with cytotoxic CTAB concentrations, the latter from 500 to 150 μM. These changes result in a more stable cell count during FI detection, which significantly increases reliability of results.

Flow cytometry allows exact quantitation of FI on a single cell basis and is considered to be superior, by accuracy, compared to FI evaluation by confocal microscopy or multi-plate reader analysis. In fact, the data obtained using confocal microscope (Figure 6) and using flow cytometer (Figure 7) display some crucial differences:

(a) The data obtained with flow cytometer are much more precise: the quantity of reads is identical for each sample (3000 reads), resulting in a negligible SD, indeed.
(b) The quantity of sulfane sulfurs in cells treated with GSH plus stimulators is over 20% higher than that detected in stimulated controls, CTRL (+), while no significant difference can be detected by microscopy.
